# Inference of weak-form partial differential equations describing migration and proliferation mechanisms in wound healing experiments on cancer cells

**DOI:** 10.1371/journal.pcbi.1013607

**Published:** 2025-10-28

**Authors:** Siddhartha Srivastava, Patrick C. Kinnunen, Zhenlin Wang, Kenneth K.Y. Ho, Brock A. Humphries, Siyi Chen, Jennifer J. Linderman, Gary D. Luker, Kathryn E. Luker, Krishna Garikipati

**Affiliations:** 1 Department of Mechanical Engineering, University of Michigan, Ann Arbor, Michigan, United States,; 2 Michigan Institute for Computational Discovery & Engineering, University of Michigan, Ann Arbor, Michigan, United States,; 3 Department of Aerospace & Mechanical Engineering, University of Southern California, Los Angeles, California, United States,; 4 Department of Aerospace Engineering, Auburn University, Auburn, Alabama, United States; 5 Department of Chemical Engineering, University of Michigan, Ann Arbor, Michigan, United States,; 6 Department of Radiology, University of Michigan, Ann Arbor, Michigan, United States,; 7 Department of Biomedical Engineering, University of Michigan, Ann Arbor, Michigan, United States,; 8 Biointerfaces Institute, University of Michigan, Ann Arbor, Michigan, United States,; 9 Department of Mathematics, University of Michigan, Ann Arbor, Michigan, United States,; Oxford, UNITED KINGDOM OF GREAT BRITAIN AND NORTHERN IRELAND

## Abstract

Cancer metastasis, which requires migration of cancer cells away from the primary tumor, is responsible for approximately 65% percent of cancer-related deaths. Therefore, targeting signaling pathways that drive cancer cell migration or proliferation is a common therapeutic approach. Cell migration is commonly studied using experimental approaches which track cells or cell monolayers as they evolve over time. Computational modeling can then be used to fit partial differential equation (PDE) models to the data, providing mechanistic insights underlying the observed cell motion, including the contribution of various cellular behaviors such as random motion, directed motion, and cell division. A popular experimental technique, the scratch assay, measures the migration and proliferation-driven cell closure of a scratch in a confluent cell monolayer. However, these assays do not disambiguate between different drivers of scratch closure (for instance between cell proliferation and migration to open space). To improve analysis of this technique, we combine scratch assays, video microscopy, and PDE inference to gain quantitative insight to mechanisms of cell migration and proliferation. We capture the evolution of cell density fields over time using live-cell microscopy and automated image processing. Our PDE inference methods involve the use of weak form-based system identification techniques for cell density dynamics modeled with advection-diffusion-reaction systems. We then compare our method with recent modeling work, finding that our model discovery tool automatically identifies similar models including reaction and diffusion terms from a larger set of bases. We demonstrate the application of this framework on 2-dimensional scratch assays subject to the inhibiting effect of trametinib on wound closure and characterize the results in the context of the quantified uncertainty in our inference approach. Our integrated experimental and computational pipeline can be used to rapidly identify and refine models of cell migration in a variety of contexts, enabling the quantitative measurement of the effect of drugs and other perturbations on cell migration and proliferation with uncertainty accounted for.

## Author summary

Collective cell migration underlies a wide range of biological phenomena, from cancer migration to tissue regeneration. Mathematical models, based on random and directed cell motion and cell birth or death, have been used to understand cell migration in a variety of contexts. However, such models can be time consuming to develop. Here, we advance a model discovery tool which rapidly and automatically identifies parsimonious models of cell migration. We validate our tool against previously analyzed data, and then deploy it to model cell migration in the presence or absence of a chemotherapeutic drug. We find that the drug reduces random cell migration by approximately 40%. We envision our tool being used to rapidly identify quantitative models of cell migration to compare the effects of new drugs or genetic perturbations.

## 1. Introduction

Cell migration is a complex, multiscale phenomenon that integrates many different inputs and cell behaviors, including directed and random motion, that are differentially regulated by signaling kinases, cell density, and other factors [1,2]. Cell migration helps maintain or form tissues or monolayers both in vivo and in vitro [3,4]; cell division also contributes to the development or maintenance of these multicellular structures. Further, cell migration is associated with cancer metastasis, and therefore is commonly studied in the context of oncogenic mutations or cancer treatments.

Inferring relationships between perturbations such as drugs or genetic modifications and migratory outputs is challenging. Such perturbations could affect one or more different drivers of cell migration, potentially in different ways, with difficult-to-predict overall effects. Cell migration is commonly measured using scratch assays, where a monolayer of cells is scratched to physically remove cells in a localized region [5]. Subsequently, migration into the newly emptied space is measured over time. These measurements are commonplace in biological research, and have, for instance, been used to identify novel inhibitors of cell migration or genes responsible for regulating migration [6,7]. However, typically the complexity of cell migration is reduced to a single number, which represents the amount of space filled in a given time or the distance traveled by the leading edge of cells [8–10]. This reduction to a single number obscures the behavioral changes that modify migration and could, for instance, fail to identify differences between drugs that inhibit migration to the same degree, but through the modulation of either directed migration or cell proliferation. Thus, the utility of scratch assays could be improved by identifying relationships between biological perturbations and specific biological effects.

Computational and mathematical modeling of cell migration has been used to extract granular, quantitative information from scratch assays. First-principles modeling has established a variety of partial differential equation (PDE) models that accurately represent the behavior of scratch assays with a high cell density and under a variety of conditions. [11,12] These models commonly include reaction terms, which represent cell proliferation or death, and diffusion terms, which represent random cell motion, with various functional forms. For example, some models use a reaction equation corresponding to cells growing with a constant proliferation rate, affected by a maximum carrying capacity. Other terms, including advection (directed cell migration), could also be included to model scenarios where cells have a directional movement stimulus, such as chemotaxis or migration to regions of lower cell density. Finally, delay differential equations have been employed to better model the observed time-dependence of cell migration in some contexts. [11,13] By estimating parameters and quantifying parameter uncertainty for these models, past work has helped identify quantitative effects of biological perturbations in scratch assays [11,13–15].

While, the development of some of the foregoing classes of models has traditionally been laborious, requiring many months, it would be desirable to rapidly and quantitatively compare behaviors from assays performed in different conditions, including experiments performed using novel drugs, in 3-D culture, [16] or in the presence of external gradients, [17,18] where previous models might not be appropriate. Modern data-driven approaches that allow for more rapid model development are discussed next.

Recent work has also established the use of physics-informed neural networks to model cell migration in scratch assays [13]. In this approach, a neural network is trained to predict the progression of cell densities over time. During training, the neural network optimizes a loss function which penalizes both inaccurate predictions and deviations from a pre-defined reaction-diffusion model. In this way, the neural network learns density-dependent proliferation and diffusion terms which best fit the data. However, in such approaches, neural networks can learn relationships to make predictions without any guarantee that the growth or diffusivities learned are physically realizable.

Another method for learning governing PDEs from experimental data and known physical constraints is Variational System Identification (VSI). It extends the popular SINDy approach [19–23] to inferring PDEs in weak form. The weak form-based inference of PDEs presented as Variational System Identification and employed in this study [24,25] was also independently developed under a different name, Weak-SINDy, introduced by Messenger and Bortz [26]. The weak form formulation is particularly useful in the presence of noise because it allows for lower-order derivatives in the governing equations, thereby reducing the amplification of noise that typically occurs when estimating higher-order derivatives from data [24,25,27]. Variational System Identification enables modelers to identify a library of physically meaningful operators (e.g, differential terms such as the gradient, Laplacian and biharmonic operators, as well as algebraic ones such as polynomials, trigonometric functions and exponential functions) which make up the PDE. Variational System Identification then identifies parsimonious models incorporating a subset of operators by iteratively dropping terms from the library. Variational System Identification and SINDy respectively use the weak and strong forms of differential equations in regression-based approaches, and therefore do not require repeated forward evaluations of the model, unlike traditional PDE-constrained inverse modeling. This substantially reduces the computational cost. Variational System Identification has been applied to identify governing equations for the evolution of materials [24,25,28], the spatiotemporal spread of COVID-19 [27,29], as well as constitutive models of soft materials [30,31]. After identifying a sparse set of operators, their coefficients in the inferred PDE can be fine-tuned using PDE-constrained optimization and validated against additional experimental test data. Finally, the surviving operators and parameters can be compared among models inferred from datasets under different conditions in order to extract quantitative insights from data.

**Fig 1:**
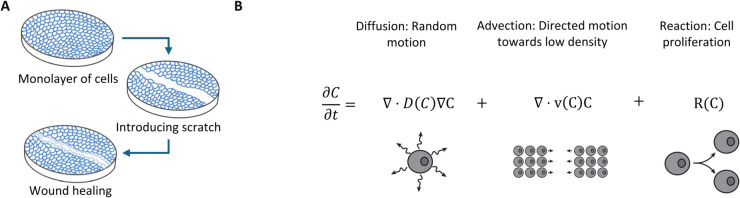
(A) Illustration of wound healing/scratch assay. (B) Diffusion, advection, and reaction mechanisms for cell random motion, directed motion and proliferation, respectively.

We hypothesized that a two-stage approach–first using Variational System Identification for rapid model discovery and then refining the inferred models using PDE-constrained optimization– could be applied to wound healing data, providing quantitative comparisons between different conditions. To test this hypothesis, we first applied the approach to previously published wound healing data [11]. With it, we were able to rapidly identify an accurate model describing the evolution of cell density over time for a variety of initial seeding densities with accuracy that matched or improved upon results in the literature [11]. Reaction and diffusion terms were identified underlying the observed behavior and consistent with previous results. We next applied Variational System Identification to our own data. We performed scratch assays on MDA-MB-231 breast cancer cells under a variety of conditions and used automated image processing to extract cell density fields over time. We found that Variational System Identification could successfully identify mechanisms of advection (directed motion), diffusion (random motion) and proliferation of cells in 2-D scratch assays by inferring the governing PDEs. These mechanisms are illustrated in Fig 1. Extending the approach’s application, we quantitatively inferred the effect of trametinib, a MAPK pathway inhibitor, on cell diffusion and proliferation. We present this work as a useful modeling approach to rapidly identify plausible models for cell population dynamics and quantify model effects under different experimental conditions.

## 2. Methods

### 2.1. Stable cell line generation

We engineered the MDA-MB-231 breast cancer cells used in this work to express a stable histone-2B (H2B) nuclear marker (mCherry), along with kinase translocation reporters [32] for the Akt and ERK kinases, as described previously [33,34]. We refer to these cells as pHAEP cells. Only the nuclear marker was used for tracking cells.

### 2.2. Cell culture

We cultured MDA-MB-231 pHAEP cells in Dulbecco’s Modified Eagle Medium (DMEM) with 10% fetal bovine serum (FBS). We passaged cells at a 1:10 ratio when they were approximately 90% confluent. For imaging experiments, we cultured cells in imaging media, consisting of fluorobrite phenol red-free medium supplemented by variable FBS (depending on desired experimental conditions), 1X penicillin/streptomycin, 1X GlutaMAX, and 1X sodium pyruvate. Sodium pyruvate was added as an antioxidant to reduce imaging-induced stress.

### 2.3. Scratch assay and live cell microscopy

For scratch assay experiments, we seeded 50,000 MDA-MB-231 pHAEP cells in 1 ml imaging media in a 24-well glass-bottom imaging plate. We grew cells to full confluency (approximately 36 hours) before starting the scratch assay. For the scratch assay, we manually scratched each cell monolayer using a p200 pipette tip. Immediately after scratching, we washed the cells with 1 ml warm phosphate buffered saline (PBS) and then added 1 ml of warm imaging media containing experimental treatments.

We imaged cells using an EVOS M7000 fluorescent microscope with on-stage incubator. After scratching each well, we placed the well plate into the prepared EVOS incubator. The incubator was maintained at 37^°^C, 5% CO_2_, and >80% humidity. For time-lapse imaging, we captured fields centered on the scratch near the center of each well. We captured fluorescence from mCherry to position individual cells over time. We acquired images every 20 minutes over 48 hours in all wells in the well plate. We imaged one region of the scratch per well. We quantified wound closure using MATLAB. We stitched the wound images together, drew a line at each side of the wound at 0 hours and 48 hours, and then calculated the distance between the lines.

### 2.4. Automated image processing

We processed fluorescent images of the MDA-MB-231 pHAEP cells as described previously [35]. Image processing was performed using MATLAB. Briefly, we first thresholded the nuclear images using an adaptive thresholding method. After identifying nuclear pixels, we extracted the centroid pixel of each distinct nuclear object. In contrast to previous work, we did not track each cell between frames, because we were interested in tracking the evolution of the density field, not individual cells. After automated image processing, we extracted cell density fields C(x,t) (number$/\mu m^{2}$) from each well in the experiment. (Here, $x=(x_{1},x_{2})\in {\mathbb{R}}^{2}$ is the two-dimensional position vector of a point.) To do so, we applied spatial binning to the cell positions. Bin sizes between 50μm and 100μm were used. We smoothed C(x,t) in space and time. We applied a moving average filter with a window of 150 μm to spatial data and a window of 3 hour and 40 minutes to temporal data. Thus smoothing the data alleviates numerical noise in the calculation of derivatives.

The bin sizes were chosen empirically to ensure that each bin contained a sufficient number of cells to meaningfully represent cell density and continuum behavior. This approach naturally led to a noisy field with discrete jumps, as cells moved between bins, necessitating additional filtering to smooth the data field for PDE inference. The filtering window sizes were determined using synthetic data with added Gaussian noise, where we identified the smallest window size that effectively reduced noise while preserving spatial and temporal variations in the data. Synthetic data generation was guided by the expected noise levels in experimental data to ensure robustness.

### 2.5. Wound healing quantification

We quantified wound closure by identifying the distance between wound edges at the first, $d(t_{\text{start}})$ and last $d(t_{\text{end}})$ time instants in the experiment. By aligning the wound edges with the *x*_2_−axis, we estimated the position of the front of each wound as a line *x*_1_ = *d*(*t*) at time *t* and calculated the distance in the *x*_1_ direction (perpendicular to the wound) between each wound front. Then, we calculated wound closure as:

$$\text{Wound closure}=\frac{|d(t_{\text{start}})-d(t_{\text{end}})|}{d(t_{\text{start}})}$$ (1)

## 3. Continuum-scale data-driven modeling for cell migration

The cell density is defined as a spatiotemporal field, C(x,t) with (x,t)∈Ω×[0,T] where $\Omega \subset {\mathbb{R}}^{2}$ and [0,*T*] are the spatial domain and time period of interest. The evolution of this field is described by the following PDE, which is of first-order in time:

$$\frac{\partial C}{\partial t}=L(C;\theta )$$ (2)

where, L is a differential operator parameterized by θ. Given θ the PDE also can be stated in terms of the residual:

$$R(C;\theta )=\frac{\partial C}{\partial t}-L(C;\theta ).$$ (3)

For some $\tilde{C}$ that is not the solution of the PDE, $R(\tilde{C};\theta )$ is the pointwise residual, equivalent to the error in satisfaction of the PDE. Obtaining the solution of the forward PDE problem means finding *C* such that R(C;θ)=0, given θ. System identification, on the other hand, is an inverse problem, in which, given data $C^{d}(x_{i},t_{j})$ at a finite number of sampling points $x_{i}$ and times *t*_*j*_ from measurements of a field representing *C*, we seek the optimal parameters $\theta^{*}$ satisfying

$$\theta^{*}=\underset{\theta \in \Theta }{\text{arg min}}\left|\;\left|\;\left|R(C^{d};\theta )\right|\;\right|\;\right|$$ (4)

where the set of admissible parameters is Θ and |||•||| is a suitable norm. The above statement of the system identification problem also holds for nonlinear PDEs. This approach to system identification reduces to a regression problem that is fairly inexpensive to solve computationally. It allows us to start with a large library of candidate terms that could comprise L and subsequently eliminate the insignificant ones using principled approaches including regularization and stepwise regression. Here, we have intentionally omitted details such as the choice of norm |||•|||, and functional spaces for $C^{\text{d}}$, in favor of presenting the basic idea of system identification. Finite element methods arrive at the residual formulated in terms of the weak form of the PDE, instead of the strong form as in (3). We adopt the weak form for PDE inference, and therefore refer to it as Variational System Identification [24,25,27]. This technique is described next.

### 3.1. Variational formulation of the advection diffusion reaction problem

The advection-diffusion-reaction equation represents transport of cell density, and is encoded in the operator, L. Mechanistically, the advection represents the directed motion of the cells, diffusion represents their random motion and reaction models cell proliferation/death. The strong form of the advection-diffusion-reaction equation is written as follows:

$$\frac{\partial C}{\partial t}=\nabla \cdot \left(D\nabla C\right)-\nabla \cdot (Cv_{f}v_{\text{unit}})+r$$ (5)

where D≡D(C) is the diffusivity, $v_{f}\equiv v_{f}(C)$ is the advective speed, $v_{unit}$ is a unit vector perpendicular to the wound, and *r* is the cell reaction rate. The statement of the Initial and Boundary Value Problem (IBVP) includes the initial density C(x,0), Dirichlet and Neumann boundary conditions on $\partial \Omega_{\text{D}}$ and $\partial \Omega_{\text{N}}$, respectively, where $\overset{―}{\partial \Omega_{\text{D}}\cup \partial \Omega_{\text{N}}}=\overset{―}{\partial \Omega }$ and $\partial \Omega_{\text{D}}\;\cap \;\partial \Omega_{\text{N}}=\emptyset$:


$$C(x,0)=C_{0}(x),\;\forall \;x\in \Omega$$

$$C(x,t)=\overset{―}{C}(x,t),\;\forall \;x\in \partial \Omega_{D},t\in [0,T]$$

$$\left(D\nabla C-Cv_{f}v_{\text{unit}}\right)\cdot n=q(x,t),\;\forall \;x\in \partial \Omega_{N},t\in [0,T]$$ (6)

The Dirichlet boundary condition specifies the cell density, and the Neumann boundary condition specifies the cell flux on the respective boundaries. Here, ***n*** is the unit outward normal vector on the boundary ∂Ω.

There exist models that have considered density-dependent effects on migration, for instance representing cells interacting with each other, and on cell proliferation, for instance, modeling its saturation at high cell density [11,36]. We incorporate such mechanisms in our model by considering parameters that are functions of cell density, *D*(*C*), $v_{f}(C)$, and *r*(*C*).

The weak form of Eq (5) is:

$$\int_{\Omega }w\frac{\partial C}{\partial t}dV=\int_{\Omega }\left(-D\nabla w\cdot \nabla C+Cv_{f}\nabla w\cdot v_{\text{unit}}+wr\right)dV+\int_{\partial \Omega_{\text{N}}}wqds$$ (7)

for all *w* in a suitable functional space, which we detail below. We seek the solution C(x,t) in the Sobolev space $H^{1}(\Omega )$, which consists of functions that are square-integrable and have square-integrable first-order weak derivatives. The field *w* is a weighting function and belongs to a subspace of $H^{1}(\Omega )$ defined as $\left\{w\in H^{1}(\Omega )\;|w=0\text{ on }\partial \Omega_{D}\right\}$.

### 3.2. The finite element form

The weak form of the advection-diffusion-reaction equation, given in Eq (7), can be discretized using the finite element method (FEM). The domain Ω is partitioned into $n_{\text{el}}$ elements, $\Omega =\cup_{e=1}^{n_{\text{el}}}\Omega_{e}$. The unknown field C(x,t) is replaced by a finite-dimensional approximation $C^{h}(x,t)$, defined over each element using a linear combination of basis functions:

$$C^{h}(x,t)=\sum_{i=1}^{n_{\text{basis}}}d_{i}(t)N_{i}(x)$$ (8)

where $d_{i}(t)=C^{h}(x_{i},t)$ are the time-dependent coefficients at finite element nodes $x_{i}$, and the spatial dependence of $C^{h}(x,t)$ is represented by the finite element basis functions $N_{i}(x)$ (also known as shape functions). The span of these basis functions defines a finite dimensional *H*^1^ space: $V^{h}=\text{span}(\{N_{i}{\}}_{i=1}^{N_{\text{\{basis}}}\})\subset H^{1}(\Omega )$. There are many ways to define these basis functions but for our study we will choose piecewise linear basis functions for one- and two-dimensional problems, the latter using triangular elements [37]. Also within the Galerkin approach, we consider finite dimensional weighting functions, $w^{h}\in \{V^{h}(\Omega )|w^{h}=0\text{ on }\partial \Omega_{D}\}$ which we write as $w^{h}(x)=\sum_{j=1}^{n_{\text{wt}}}b_{j}N_{j}$ where *N*_*j*_ are the $n_{\text{wt}}\leq n_{\text{basis}}$ basis functions of *V^h^* that vanish on $\partial \Omega_{D}$. Substituting these finite-dimensional approximations *C^h^* and *w^h^* for *C* and *w*, respectively, in (7) and invoking its validity for all $w^{h}\in V^{h}$ we obtain the following residual vector:

$$R(C^{h};\theta )=\int_{\Omega }\left(\frac{\partial C^{h}}{\partial t}Nd\Omega +D\nabla N\cdot \nabla C^{h}-C^{h}v_{f}v_{\text{unit}}\cdot \nabla N-rN\right)dV-\int_{\partial \Omega_{T}}Nqds$$ (9)

where N is the vector of finite element basis functions, and the second integral on the right imposes the Neumann boundary condition. The field *C^h^* is used to estimate the spatial derivatives in (9), as $\nabla C^{h}=\sum_{i}d_{i}\nabla N_{i}$ and the time derivative as $\frac{\partial C^{h}}{\partial t}=\sum_{i}\frac{\partial d_{i}}{\partial t}N_{i}$ where the $\frac{\partial d_{i}}{\partial t}$ is estimated using backward Euler scheme on the time-discretized data point. The residual vector $R(C^{h};\theta )$ is a finite-dimensional version of the residual of the weak form of the PDE.

### 3.3. Inference of the advection diffusion reaction system

We infer the advection-diffusion-reaction system by posing it as a standard optimization problem using the residual derived in the previous section and the cell density field from wound healing experiments (see Sect 2.4). The density field extracted from the data is written by interpolation of its values *d*_*i*_(*t*) by positioning finite element nodes $x_{i}$ such that $d_{i}(t)=C^{h}(x_{i},t)$. This allows the use of (8) with the nodal values *d*_*i*_(*t*) defining the density field $C^{h}(x,t)$. We impose Dirichlet boundary conditions on all edges. Therefore, the Neumann boundary integral of (9) does not feature in the following discussion.

We consider the following ansatz for the parameters *D*, $v_{f}$ and *r*:

$$D=\theta_{0}\cdot 1+\theta_{1}C+\theta_{2}C^{2}$$ (10a)

$$v_{f}=\theta_{3}\cdot 1+\theta_{4}C+\theta_{5}C^{2}$$ (10b)

$$r=\theta_{6}C+\theta_{7}C^{2}$$ (10c)

where *C* is replaced by *C^h^* from Eq (8) during inference. The constant reaction term was omitted, since it represents cell proliferation (*r* > 0) or death (*r* < 0) in a region with zero cell density, both of which are unphysical. We included advection terms, even though they have not previously been required to model wound closure, to demonstrate the utility of our method as a model discovery tool, and because advection has been used in other cases to model cell migration tracked with live-cell microscopy [16,17]. We also omitted delay differential terms for modeling the advection diffusion reaction system from the main focus of this work, even though they have been shown to provide slightly better fits to data in the past. [13] Although delay differential terms can be treated within the Variational System Identification framework, they pose significant challenges for PDE-constrained optimization, which is a critical subsequent component of our workflow. The combination of delay differential equations with PDE-constrained optimization remains an open area of computational research beyond the scope of this communication. Instead, in S2 Appendix we present a restricted delay model within the Variational System Identification framework, and show that its performance is comparable to that of the non-delay treatment.

We can now rewrite the residual equations equation as a matrix-vector problem with the following form, where *A* represents the standard finite element assembly operation that maps local element contributions to the global system of equations [37].

$$R(C^{h};\theta )=y-\Xi \cdot \theta$$ (11)

$$y=\underset{e}{A}\left[-\int_{\Omega_{e}}\frac{\partial C^{h}}{\partial t}Nd\Omega \right]$$ (12)

$$\Xi \equiv \left[\Xi_{0},\cdots ,{\text{Ξ}}_{7}\right].$$ (13)

Here, $N=\{N_{k}\}$ represents the vector of basis functions for $k=1,\ldots n_{\text{np}}$ where $n_{\text{np}}$ is the number of nodes in the problem, and Ξ is a matrix where the columns $\Xi_{j}$ represent the residual vector terms corresponding to diffusive, advective and reaction operators in weak form from Eq (9). The rows correspond to the components of the basis function vector N concatenated over timesteps. We have,

$$\left[\Xi_{0}\;\Xi_{1}\;\Xi_{2}\right]=\underset{e}{A}\left[\int_{\Omega_{e}}\left[1\;C^{h}\;C^{h^{2}}\right]\nabla N\cdot \nabla C^{h}d\Omega \right]$$ (14a)

$$\left[\Xi_{3}\;\Xi_{4}\;\Xi_{5}\right]=\underset{e}{A}\left[-\int_{\Omega_{e}}\left[1\;C^{h}\;C^{h^{2}}\right]C^{h}v_{\text{unit}}\cdot \nabla Nd\Omega \right]$$ (14b)

$$\left[\Xi_{6}\;\Xi_{7}\right]=\underset{e}{A}\left[-\int_{\Omega_{e}}\left[C^{h}\;C^{h^{2}}\right]Nd\Omega \right]$$ (14c)

Here, the integration over the element domain is numerically approximated using the Gauss-Legendre quadrature [37]. We finally state the inference problem for the optimal parameters as the following minimization problem with a quadratic cost defined as the Euclidean norm |R|:

$$\theta^{*}=\underset{\theta }{\text{arg min}}|R(C^{h};\theta ){|}^{2}$$ (15)

In Variational System Identification, spatial derivatives on the data field are limited to first order, despite originating from a second-order differential equation. In the weak form, derivatives that act on the trial solution in the strong form are transferred to the weighting functions, reducing the need for direct computation of higher-order derivatives. Since noise propagation in numerical differentiation scales as $𝒪(h^{-d})$ for a d-order derivative, transitioning from a second-order derivative in the strong form to a first-order derivative in the weak form reduces noise amplification by $𝒪(h^{-1})$. This significantly improves the reliability of estimated parameters in the presence of noisy data.

In the interest of model parsimony, we seek to estimate the most significant terms given ansatz Eq (10) for the parameters using stepwise regression [24,25,27]. Since an increase in parsimony comes at the cost of an attendant growth in the loss between model iterates, we adopt a search across surviving terms and select for elimination the candidate that, when excluded from the basis, leads to the minimal growth in the loss of the reduced optimization problem from Eq (15). This results in a parameter vector $\overset{―}{\theta }$ that is sparse in the sense that most of its components ${\overset{―}{\theta }}_{m}=0$ for m∈{0,…,7}.

### 3.4. PDE constrained optimization for model refinement

Following Variational System Identification that identifies the parsimonious governing PDE structure, PDE-constrained optimization is employed to refine the numerical values of the coefficients through adjoint-based gradient computations, further improving model fit. The optimization problem statement is:

$$\text{Find }\theta^{**}=\underset{\overset{―}{\theta }}{\text{arg min}}\ell (\overset{―}{\theta };C^{\prime^{h}}),\;\text{where }\ell (\overset{―}{\theta })=||C^{\prime^{h}}(x,t;\overset{―}{\theta })-C^{h}(x,t)|{|}_{L_{2}(\Omega \times [0,T])}^{2}$$ (16a)

$$\text{such that }R(C^{\prime^{h}};\overset{―}{\theta })=0$$ (16b)

$$\text{for }C^{\prime^{h}}(x,t)=\sum_{i=1}^{n_{\text{basis}}}d_{i}^{\prime }(t)N_{i}(x)\;\text{defined over each element}$$ (16c)

Here, $C^{\prime^{h}}$ is a finite element interpolant field that is different from the data-derived field *C^h^* introduced in equation 8. Thus, the optimization problem in (16) involves solution of the forward model, which can be computationally expensive and require exploring regions of parameter space that are numerically unstable, making it unsuitable for large models. However, if a parsimonious model has been identified, then the approach in (16) can be applied to refine the PDE parameters starting from their values $\overset{―}{\theta }$ inferred by Variational System Identification as initial iterates. We solve the forward model in (16b) using experimentally observed initial conditions, generating a predicted cell density field, which enables computation of an associated loss in (16a). However that minimization problem requires computing a variational derivative through the chain rule which also involves evaluating the derivative of $C^{\prime^{h}}$ with respect to $\overset{―}{\theta }$. We complete this step via adjoint-based gradient optimization with the BFGS solver. PDE-constrained optimization with adjoint-based gradient computation does not change the selected operators but generates a new set of parameter values, which fit the data better than the those obtained by Variational System Identification with stepwise regression.

### 3.5. Computational framework for inference

The numerical examples presented in this work have been posed and solved in two dimensions by the finite element method implemented on the FEniCS platform [38,39]. The 1D forward solutions presented in this work were carried out on a uniform mesh with 42 piecewise linear elements. The 2D solutions were obtained on a rectangular domain using a structured mesh with 6478 linear triangular elements. The nonlinear optimization for minimizing (15) was carried out using the *Newton solver* in the FEniCS package. The PDE constrained optimization (16) was implemented in the dolfin-adjoint package [40,41]. In the computational studies presented in the next section, for the nonlinear PDE solver, we used a Newton solver with absolute and relative error tolerance stopping criteria of 1*e*–8 and 1*e*–9 respectively. For dolfin-adjoint we used ‘L-BFGS-B’ with tolerance of 1*e*–9.

It is also worth noting the possible non-uniqueness of the identified model. In general, the ability to uniquely recover PDE parameters depends on the richness of the data, particularly the initial and boundary conditions as well as the spatiotemporal resolution of observations. Given a specific choice of initial conditions, the available simulation data may not contain sufficient information to uniquely determine all mechanisms in the PDE, potentially leading to non-uniqueness in the inferred parameters. In our previous work [42], we demonstrated that while this inference technique can successfully recover a PDE model whose solution closely matches the ground-truth simulation, the inferred parameters may differ from the true values. This suggests that while the model captures the essential dynamics of the system, multiple parameter sets may yield similar macroscopic behavior. In such cases, it is important to assess whether the inferred parameters are biophysically reasonable and consistent across different experimental conditions. In Sect 4.2, we will use the sensitivity plots to determine the degeneracy in the inferred model. From a more abstract perspective, however, it is also possible for non-uniqueness to be intrinsic to the model itself, regardless of data quality or resolution, as highlighted in recent works on structural identifiability of PDE models [43]. These studies distinguish between globally and locally structurally identifiable models, and our framework, which is linear in parameters, falls under this setting. While our VSI formulation guarantees a unique global minimum under a positive definite quadratic form, subsequent PDE-constrained optimization explores only local neighborhoods and may therefore overlook other admissible solutions.

## 4. Results

### 4.1. Variational system identification of 1-D wound healing dynamics from data

We first tested Variational System Identification on a previously reported scratch assay dataset. The dataset, collected by Jin et al., [11] consists of scratch assays performed on PC-3 prostate cancer cells with varying initial densities prior to the scratch. Different extents of confluence were achieved by varying the initial seeding between 10,000 and 20,000 cells in steps of 2000. The high cell densities that were achieved in this experiment justified the consideration of continuum advection-diffusion-reaction PDEs to model these data. For each of the resulting six datasets, cell density was measured every 12hr for 48hr and averaged across three replicates. Next, the density was averaged along the (*x*_2_) direction parallel to the scratch, yielding a one-dimensional density field (along *x*_1_) for each initial density.

**Fig 2:**
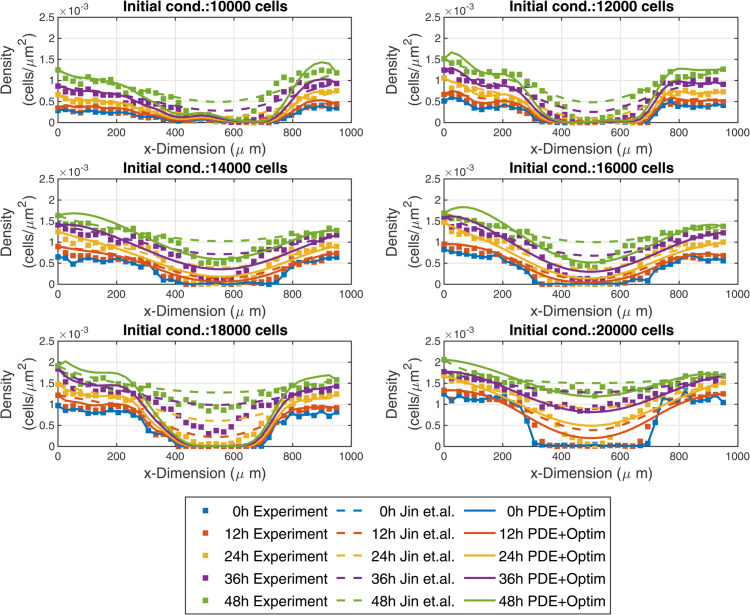
Experimental wound healing data (symbols), the model inferred by Jin et al. [11] (dashed curves), and the model inferred by Variational System Identification and refined by PDE-constrained optimization (solid curves). For the 18000 initial density data, the 4-term model was used. Inferred model parameters appear in Table 1.

The 1D cell density profiles over time are shown in Fig 2. The scratch, which is approximately 400μm long, is clear in the initial density profiles. For all initial seeding with 14000 or more cells, the center of the scratch (around $x_{1}=500\mu m$) is occupied by cells after 36 hours. Furthermore, there is an increase in cell density at the scratch edge (near x=0μm or ~950μm) over time for all initial seeding densities, indicating that the cells continue growing during the experiment.

**Table 1:** Model terms learnt by Variational System Identification and PDE-constrained parameter optimization for each experimental condition presented in Jin et al. As in the text, *C* is the cell number density measured in the units of cells/$\mu m^{2}$.

Initial density	Method	Diffusivity ($\mu m^{2}/hr$)	Reaction Term (1/*hr*)
10000	VSI inferred	$7.9+9.0\times 10^{3}C$	$3.6\times 10^{-2}C$
	VSI+Optim	$7.9+8.9\times 10^{3}C$	$2.7\times 10^{-2}C$
12000	VSI inferred	$15+2.8\times 10^{3}C$	$2.7\times 10^{-2}C$
	VSI+Optim	$15+2.9\times 10^{3}C$	$2.3\times 10^{-2}C$
14000	VSI inferred	$17+7.1\times 10^{3}C$	$2.1\times 10^{-2}C$
	VSI+Optim	$4.6\times 10^{2}+0.25C$	$2.3\times 10^{-2}C$
16000	VSI inferred	$13+1.3\times 10^{2}C^{2}$	$1.9\times 10^{-2}C$
	VSI+Optim	$3.6\times 10^{2}+1.3\times 10^{2}C^{2}$	$2.3\times 10^{-2}C$
18000 (3 Term)	VSI inferred	$17+1.3\times 10^{-3}C$	$1.7\times 10^{-2}C$
	VSI+Optim	$17+1.3\times 10^{-3}C$	$1.5\times 10^{-2}C$
18000 (4 Term)	VSI inferred	$17+1.3\times 10^{-3}C$	$1.7\times 10^{-2}C+1\times 10^{-8}C^{2}$
	VSI+Optim	$36.4+1.2\times 10^{-2}C$	$9.5\times 10^{-2}C-69.7C^{2}$
20000	VSI inferred	$22+3.1\times 10^{-2}C^{2}$	$1.2\times 10^{-2}C$
	VSI+Optim	$8.1\times 10^{2}+3.1\times 10^{-2}C^{2}$	$1.7\times 10^{-2}C$

**Fig 3:**
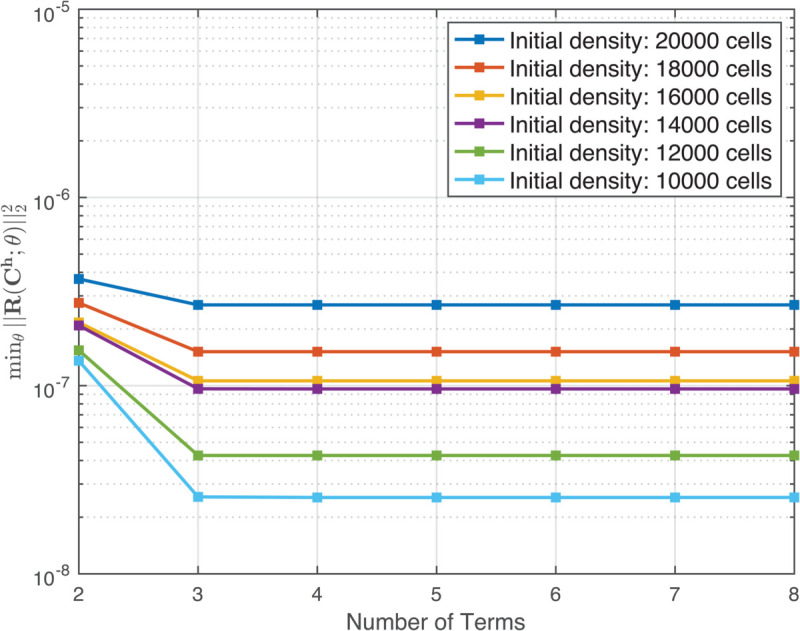
Elbow curve illustrating the Variational System Identification (VSI) loss at each step of stepwise regression. At each step, the loss is computed as the minimum residual norm over the space of all admissible parameters for the current model.

Next, we used our computational pipeline to find parsimonious models for each of the six scratch assay datasets corresponding to different initial cell densities. First, we used the data $C^{h}(x_{1},t)$ to generate diffusivity, advective velocity, and reaction rates that are polynomial functions of density up to second order (quadratic in (10)). Then, we used VSI to identify models from the generated bases. Fig 3 shows that for all cases initial cell densities, the loss does not decrease for models having more than 3 terms. In every case, the three-term model excluded advection. We next performed PDE-constrained optimization of the 3-term model for each of the six cases with different initial number of cells seeded, with the additional physical constraint of positive diffusivities. The diffusivity in each resulting optimized model has a constant term, and either a linear or quadratic dependence on local density, *C*. All the inferred reaction functions have linear density dependence, except the 4-term model for 18,000 cells (see Table 1). For the case of 18,000 cells, we observed minimal improvement in the cost function Eq (16c) from the PDE-constrained optimization. The model, both before and after refinement, is presented in Table 1 under the label 18,000 (3 terms). Furthermore, we noticed that in the 3-term model, the partial derivatives of the cost function with respect to the diffusivity coefficients remained low, indicating that the available data was not sufficiently sensitive to the diffusive mechanism of random cell motion. To address this issue, we expanded the model to include four terms–incorporating both linear and quadratic dependence on the local cell density. This expansion led to a significant reduction in the cost function during PDE-constrained optimization. The refined model, presented in Table 1 under the label 18,000 (4 terms), demonstrates a strong agreement between the model solution and the experimental data. The diffusivity values after refinement by PDE-constrained optimization have a constant term and a positive linear or quadratic dependence on the local cell density, *C*. This suggests a mechanism of intercellular communication between the cells, which is likely to be contact-mediated, and drives the cells to further explore their immediate vicinity by random walks. However, given that the maximum cell density satisfies $C\leq 2\;\times \;10^{-3}\;\mu {\text{m}}^{-2}$ (Fig 2), this density dependence is weak. The constant diffusivity term has a roughly increasing trend with the initial number of cells seeded (except for the outlier at 18,000 cells that interrupts this increasing trend, Table 1). The trend for higher diffusivity with increase in the number of cells initially seeded is consistent with the increase of diffusivity with local density *C*. The proposed mechanism of contact-mediated random walk explorations of the environment is also a reasonable explanation for both aspects of increasing diffusivity. Similarly, refinement of the inference results by PDE-constrained optimization yields a reaction (proliferation) term with a weak linear dependence on local cell density *C* for all except the 18,000 cell seeding case. Note that the final form of the reaction (proliferation) with the 4-term 18,000 cell model has a negative quadratic dependence on density that results in a decrease of proliferation at higher densities.

**Fig 4:**
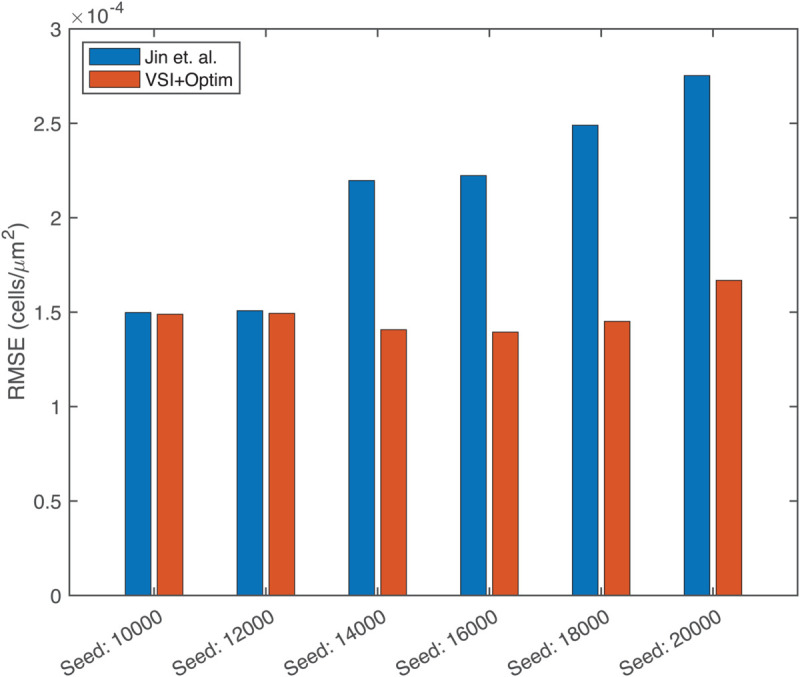
Root mean squared error (RMSE) calculated between experimental data and reaction-diffusion models from Jin et al. (blue), and a model obtained by PDE-constrained optimization following Variational System Identification (red) for each case with different initial number of seeded cells. For the 18000 initial density data, the 4-term model was used. The model parameters inferred in this work are shown in Table 1.

We used these refined models to run a forward simulation from the initial conditions of each dataset with the different numbers of initial cells seeded. The model predictions and experimental observations appear in Fig 2, where we also present the forward simulation of the reaction-diffusion model using the parameters provided by Jin et.al. [11]. We draw the reader’s attention to the qualitative match between the forward solutions and the dynamics observed in the dataset at early times and the quantitative match at later times. We found that for four of the six initial seeding densities, our models have root mean squared errors (RMSEs) that are significantly lower than the RMSE reported by Jin et al. and are similar to their results for the other two cases (Fig 4). These results suggest that our inference framework of Variational System Identification and PDE-constrained parameter optimization can be used to infer models for cell migration dynamics that are competitive with previous methods such as those by Jin et al, which fit a model based on prior knowledge about the system. In this work, we considered a polynomial cell density dependence in the diffusivity for modeling nonlinear diffusion, but at higher cell densities, more rigorous models, such as Maxwell-Stefan diffusion would be more appropriate [42]. In such formulations, phenomenological extensions can be constructed by treating void space as an additional species, which effectively yields a single-independent-component representation of nonlinear diffusion.

### 4.2. Model-based quantification of the effect of inhibitor levels on migration dynamics in 2-dimensional scratch assays

**Fig 5:**
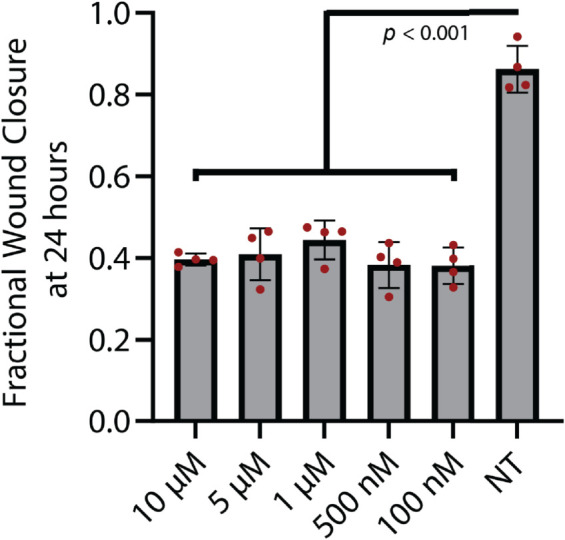
Fractional wound closure Eq (1) calculated for a range of trametinib concentrations and an untreated control (labeled NT), with *N* = 4 wells for all conditions. Individual datapoints are shown in red, and the mean +/- standard deviation is shown by the bars and error bars, respectively.

**Fig 6:**
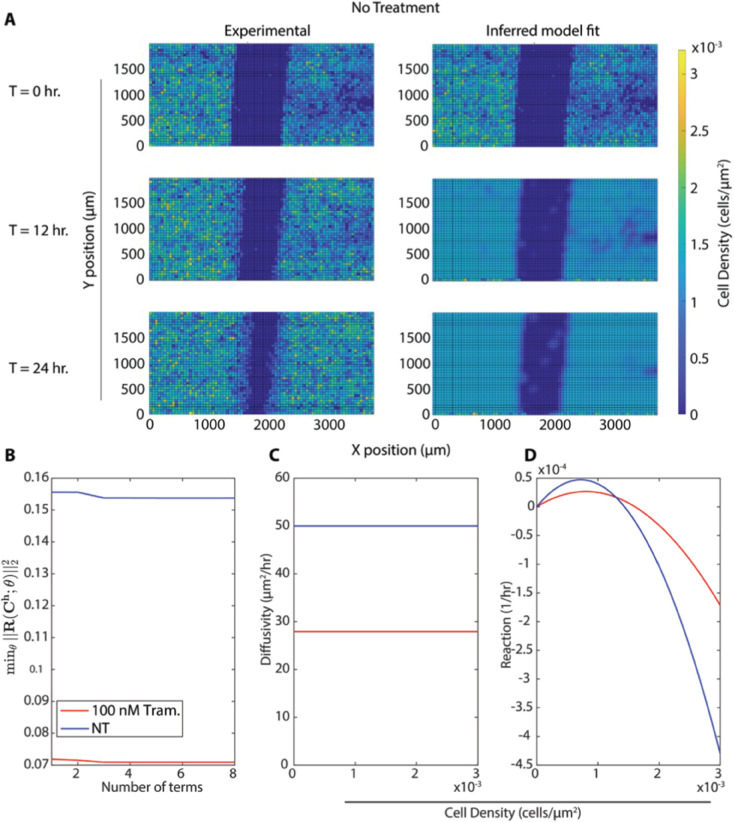
(A) Comparison of experimental data and predictions of a model inferred by Variational System Identification followed by PDE-constrained parameter optimization under the same initial conditions. The experimental condition is with no treatment. (B) Variational System Identification loss as a function of the number of terms in the model inferred for the untreated and 100 nM trametinib conditions. (C-D) PDE-constrained and optimized diffusivity (C) and reaction (cell proliferation) (D) terms as functions of density. Diffusivity is constant, while reaction/proliferation is a combination of linear and quadratic terms. The horizontal axis ranges were chosen to represent the densities present in the experimental data.

After demonstrating that Variational System Identification and PDE-constrained optimization could be used to learn parsimonious advection-diffusion-reaction models for cell migration data in the literature, we used the approach on our own cell density data gathered from fluorescence microscopy experiments. We performed scratch assays using MDA-MB-231 breast cancer cells and tracked scratch closure over time using live-cell fluorescence microscopy Fig 5. As seen in Fig 6A, the cell densities achieved are comparable to those observed by Jin et al., [11] and admit treatment of the problem using continuum advection-diffusion-reaction PDEs.

We used this assay to compare how trametinib, a MEK kinase inhibitor, affects cell migration. MEK kinase is known to regulate cell migration and proliferation [44]. Our past study showed that trametinib reduces both random and directed motion in chemotaxis [18]. We captured wound-closure dynamics for cells exposed to 5 trametinib concentrations from 10 *μ*M to 100 nM, and for an untreated control. We observed that untreated cells were able to almost close the wound after 24 hours, while 100 nM trametinib was enough to significantly inhibit wound closure. Higher trametinib concentrations did not further inhibit wound closure (Fig 5).

**Fig 7:**
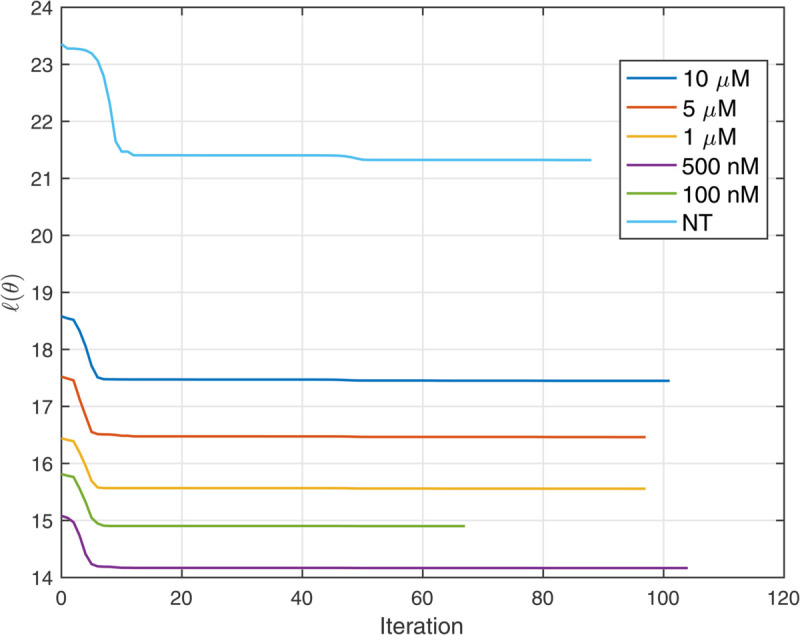
Loss minimization during PDE-constrained optimization process.

**Fig 8:**
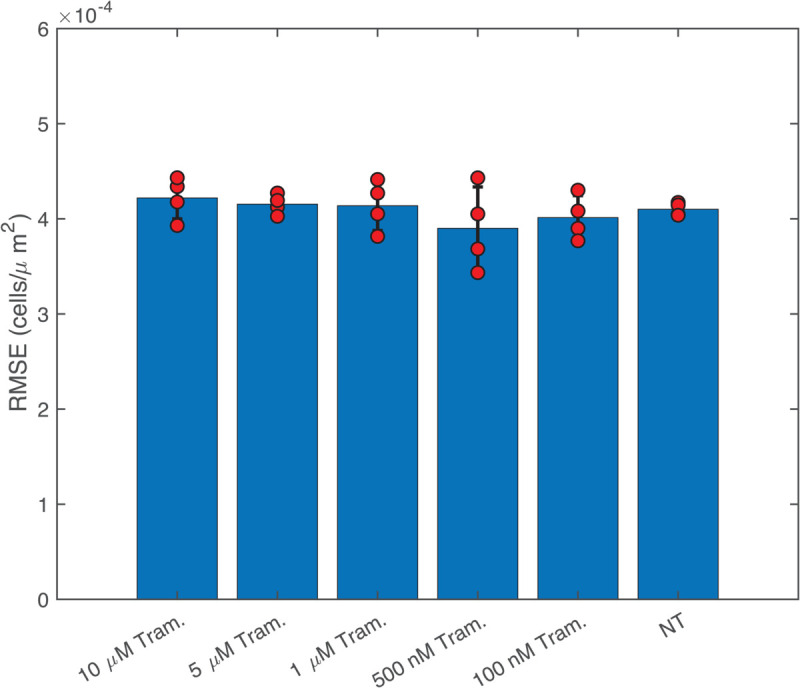
RMSE evaluated between the forward prediction of VSI+Optim models (Variational System Identification and PDE-constrained parameter optimization) presented in Table 2. The red dots represent the RMSE for each replicate, and the bar represents the mean value.

We next applied our PDE inference pipeline to learn models for the observed wound closure dynamics. We used the same library of candidate operators, including constant, linear, and quadratic terms for diffusion and advection, and linear and quadratic terms for reaction. Four replicates of the scratch assay were obtained for each treatment condition (five trametinib concentrations and one with no treatment). The fractional wound closures across the replicates for each treatment condition had standard deviations less than 15% of the mean (Fig 5). Guided by this reproducibility, the PDE parameters were inferred using the data aggregated across replicates, generating a single model for each condition. We aggregated the data by minimizing the VSI loss function (equations 15 and 16) across all 4 replicates. The PDE-constrained minimization procedure results in further refinement of losses as shown in the Fig 7. The average loss and individual losses for each replicate are presented in Fig 8, indicating consistent losses across replicates. This verifies that the inferred PDE model does not exhibit sensitivity to initial conditions, such as distribution of cells, which would vary between the replicates. Finally, we ran the inferred model for each condition starting from the experimentally measured initial cell density (Fig 6A). We observed that the Variational System Identification loss function is essentially constant for two-term models including diffusion and reaction terms, and only starts to increase for models that lack reaction terms (Fig 6B). Based on the these results, we adopted a three-term model. For both the untreated condition and 100 nM trametinib, these terms corresponded to constant (density-independent) diffusivity, and reaction terms that are linear and quadratic in cell density. Table 2 shows the coefficients inferred initially by Variational System Identification in the process of delineating mechanisms, and their subsequent refinement by PDE-constrained optimization. After model refinement, our inferred models show that diffusivity decreases by approximately 44%, from $50\mu m^{2}/hr$ in the untreated cells to $28\mu m^{2}/hr$, in 100 nM trametinib, and remains comparable for higher levels of the inhibitor. We also found a non-linear functional dependence of cell proliferation (reaction in the PDE) rate on cell density: positive at low cell densities and negative above a critical density; i.e., the model predicts cell death sets in (Fig 6D). This inferred mechanism could be interpreted as a crowding-induced death in the cell population. However, no cell death was observed in our experimental data. The models inferred with and without trametinib suggest that even 100 nM trametinib causes a decrease in random cell migration and cell proliferation. Notably, the effect of trametinib treatment quickly saturates, as is reflected in the coefficients for constant diffusivity and linear/quadratic reaction terms in Table 2. The RMSEs between the forward predictions of the model and the data are presented in Fig 8, and are essentially comparable across trametinib concentrations and for the untreated case.

**Table 2:** Model terms inferred by Variational System Identification and PDE-constrained parameter optimization for each experimental condition shown in Fig 3. As in the text, *C* is the cell number density measured in the units of cells/$\mu m^{2}$.

Condition	Method	Diffusivity ($\mu m^{2}/hr$)	Reaction Term (1/*hr*)
10 μM	VSI inferred	6.8	$5.5\times 10^{-2}C-3.4\times 10^{1}C^{2}$
	VSI+Optim	$2.7\times 10^{1}$	$8.2\times 10^{-2}C-5.2\times 10^{1}C^{2}$
5 μM	VSI inferred	7.2	$5.0\times 10^{-2}C-3.0\times 10^{1}C^{2}$
	VSI+Optim	$2.8\times 10^{1}$	$7.0\times 10^{-2}C-4.4\times 10^{1}C^{2}$
1 μM	VSI inferred	6.2	$5.2\times 10^{-2}C-3.2\times 10^{1}C^{2}$
	VSI+Optim	$2.3\times 10^{1}$	$7.1\times 10^{-2}C-4.5\times 10^{1}C^{2}$
500nM	VSI inferred	7.8	$4.8\times 10^{-2}C-2.9\times 10^{1}C^{2}$
	VSI+Optim	$3.0\times 10^{1}$	$6.3\times 10^{-2}C-4.0\times 10^{1}C^{2}$
100 nM	VSI inferred	7.3	$5.1\times 10^{-2}C-3.0\times 10^{1}C^{2}$
	VSI+Optim	$2.8\times 10^{1}$	$6.6\times 10^{-2}C-4.1\times 10^{1}C^{2}$
NT	VSI inferred	9.1	$9.1\times 10^{-2}C-6.0\times 10^{1}C^{2}$
	VSI+Optim	$5.0\times 10^{1}$	$1.3\times 10^{-1}C-9.2\times 10^{1}C^{2}$

**Fig 9:**
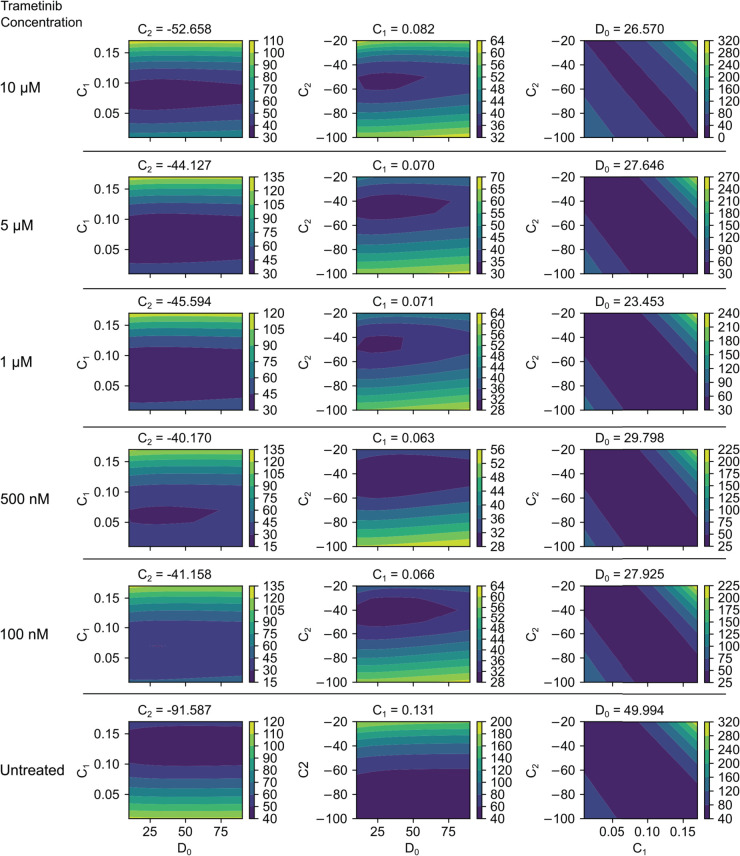
Sensitivity analysis of the inferred parameters in the 3-term model: $\partial C/\partial t=D_{0}\Delta C\;+\;C_{1}C\;+\;C_{2}C^{2}$. Contour plots show the normalized loss function from Eq (16a) as a function of parameter variations. Each column represents a case where one parameter– *C*_2_ (left), *C*_1_ (middle), or *D*_0_ (right)–is held fixed at its inferred value, while the remaining two parameters are varied in a neighborhood around their inferred values: $\left(D_{0},C_{1}\right)$, $\left(D_{0},C_{2}\right)$, and $\left(C_{1},C_{2}\right)$, respectively. Each row corresponds to one of the six experimental conditions. Contours illustrate the sensitivity of the loss function to changes in the inferred parameters, where sharp minima indicate high sensitivity (well-constrained parameters), and broader regions suggest lower sensitivity (potential parameter degeneracy).

To assess the sensitivity and robustness of the inferred parameters in the 3-term model, we analyze the contour plots of the loss function (16a) as a function of parameter variations in Fig 9. In each plot, one parameter is held fixed at its inferred value, while the remaining two are systematically varied. The first two columns reveal a shallow minimum in the diffusion parameter direction, suggesting that a broad range of admissible diffusive values can yield similarly low losses, indicating lower sensitivity to diffusion estimates. The third column highlights an affine relationship between the coefficients of the linear and quadratic reaction terms, showing a lack of sensitivity along this direction. It can be shown that the ratio $-C_{2}/C_{1}$ is equal to the carrying capacity (the maximum cell density) used in previous work [11]. The linear relationship between *C*_2_ and *C*_1_ shows that while the carrying capacity is sharply estimated, the growth rate exhibits greater uncertainty.

We note that our current analysis uses point estimates of the inferred parameters, which does not provide a complete picture of the underlying parameter distributions. If the posterior distribution of a parameter is broad or flat, then comparing single values, whether they are maximum likelihood estimates (MLEs), maximum a posteriori (MAP) estimates, or other point summaries, can obscure the true level of uncertainty. A more comprehensive characterization in terms of posterior means, variances, correlations, and higher moments would provide a deeper understanding of how drug treatments influence the parameters.

A full Bayesian treatment using Markov Chain Monte Carlo would represent a rigorous approach to obtain such posterior distributions. However, for PDE models with repeated forward evaluations, especially in the presence of the high variability suggested by our sensitivity plots, this becomes computationally expensive, requiring a large number of proposed points to adequately explore the parameter space. In place of such a Bayesian treatment, which we are pursuing in related work, we have performed a post-inference variability analysis of the inferred parameters using a local Gaussian approximation to the posterior in S1 Appendix. As that analysis shows, the standard deviation on the inferred diffusivity is of the order of the reported parameter values. This large uncertainty reflects the nature of random motion and the second-order Laplacian of the concentration which amplifies noise in the data as we have shown previously. [24] Given the analysis in S1 Appendix, the ~44% decrease in diffusivity upon application of 100 nM trametinib that we report in Table 2 corresponds to one parameter set that explains the data, but other combinations with smaller differences in diffusivity also could be admissible models.

## 5. Discussion

The treatment of cell migration via continuum PDEs is well established in biophysics [45–47], as is the role of cell arrangement in determining aspects of the progression of cancers [48]. Here, we demonstrate the strength of our two-stage inference approach, where Variational System Identification (VSI) rapidly identifies candidate PDE structures, and PDE-constrained optimization further refines the inferred parameters, enabling the discovery of parsimonious, quantitative, and physics-based models for collective cell migration. In a 1D setting, we infer models with accuracies that are competitive with other recent approaches. We also have applied our approach to our own 2D wound healing experiments. Relative to the 1D data, our 2D wound healing experiments were sampled more frequently (every 20 minutes compared to every 12 hours) with similar spatial resolution. In this setting, we estimate diffusivity values for breast cancer cells around 50 $\mu m^{2}/hr$ in untreated conditions, decreasing to between 46−60% of that value in response to trametinib. Previous computational analyses of cell migration, conducted on data gathered from a variety of experimental cell lines, have identified a wide range of diffusivity values, from approximately 50 to 3000 $\mu m^{2}/hr$ [12,49,50]. Past work focused on U251 glioma cancer cells measured a random motion coefficient (akin to diffusivity measured for a single cell) of between 60 and 300 $\mu m^{2}/hr$ across a range of substrate stiffnesses [51]. Hence, our estimated diffusivity values for both PC-3 prostate cancer cells [11] and MDA-MB-231 breast cancer cells are consistent the lower end of the range of prior measurements and close to values measured previously in other cancer cell lines. We note, however, that the uncertainty in diffusivity that emerges in the post-inference variability analysis of the parameters in S1 Appendix shows that other parameter combinations also could explain the trametinib results.

There are some advantages to inference by Variational System Identification followed by PDE-constrained parameter optimization compared to other approaches such as traditional model inference or Physics Informed Neural Networks (PINNs). Traditionally, building models of cell migration has utilized iterative cycles of model conceptualization, based on known cell biology, that include increasingly complex models for cell division or diffusion. These models are informative and have made substantial progress towards a general theory of collective cell migration. However, model development can require a substantial amount of expertise or trial and error. Furthermore, this approach does not scale well if new sources of data are acquired, which could increase the number of potential model terms based on the interaction of a new data source with all other types of data previously considered in the model. For example, data about cell state (e.g. signaling activity, cell-cycle status, metabolic activity) gathered from fluorescent reporters or cell morphology could require updating a model to consider diffusivity as a function of both cell state and local cell density [3,52–54]. Hence, we envision that Variational System Identification and PDE-constrained parameter optimization can improve traditional cell dynamics and signaling modeling in at least two ways. First, it could serve as a hypothesis-testing tool. When considering a library of mathematically expressed candidate mechanisms and datasets to test against, modelers could use the approach advanced here to develop models that explain the data within specified error bounds and with a desired parsimony. Second, the approach presented here could identify new experimental conditions to generate data that activate mechanisms not queried by existing datasets. Traditional modeling can be time consuming, and a more rapid, semi-automated model testing approach such as Variational System Identification combined with PDE-constrained parameter optimization could thus lead to more tightly coupled model-driven experimentation and data generation.

We also envision two key applications of Variational System Identification followed by PDE-constrained parameter optimization furthering our understanding of biology more broadly. First, it can be used to quantify the effects of drugs in high-throughput screens. Scratch assays have been miniaturized and mechanized, making them compatible with high-throughput screening [55,56]. Thus, the approach presented here with trametinib could be extended and combined with high-throughput drug screens so that the specific effects of drugs on cell migration and division could be determined. Second, our approach could be used to rapidly infer models based on new streams of data gathered from scratch assays. Cell morphology [52] and fluorescent reporters [54] have been used to measure or infer cell states in migrating cells. Thus, Variational System Identification followed by PDE-constrained parameter optimization could be used to resolve diffusivity or reaction (proliferation) terms as functions of not only cell density but also local measures of cell state.

This approach identifies the best-fit PDE for the data within the space of models represented by the chosen basis functions for different mechanisms. Since this is a mechanism-based model, it can generalize beyond the experimental conditions to some extent. However, there are inherent limitations to such extrapolation. In certain cases, particularly at lower cell densities, some mechanisms may not be activated, leading to their exclusion during inference. A similar issue arises with the inferred quadratic terms in the reaction function during the VSI procedure. A positive coefficient suggests a superlinear increase in local cell density at low densities, which is unlikely to persist at higher densities due to biological constraints. However, since the dataset does not contain sufficiently high-density regions, the PDE inference process lacks the necessary information to constrain the quadratic term appropriately. While one could impose a negative coefficient constraint on the quadratic term to enforce saturation effects at high densities, doing so might completely ignore the observed superlinear growth at low densities, which would also be non-physical. This highlights the trade-off between model flexibility and prior biological knowledge in the PDE inference process. A potential solution to this issue is expanding the candidate function space for the inferred PDE. In this work, we have chosen a simple polynomial basis, however, more sophisticated functional forms [57] could lead to more biologically robust models. We will explore these extensions in future studies to improve model generalizability while maintaining mechanistic interpretability.

We draw attention to the fact that the advective terms consistently dropped out during the VSI procedure because their inclusion did not contribute to reducing the loss functional. This outcome reflects that, within the spatio-temporal resolution of the wound healing experiments considered here, advective transport was not detected as a dominant mechanism. We would like to emphasize, however, that this result does not imply the absence of advective influences in general. Rather, it indicates that any such effects were not detectable at the timescale and under the experimental conditions analyzed in this study. Our initial motivation for including advective terms in the candidate library was to allow for the possibility of directed migration behavior. While these effects may not have been resolvable in the present dataset, the framework remains capable of capturing advective transport if it becomes significant under different experimental conditions (for example, alternative initial conditions, boundary conditions, or chemical perturbations). While our current results suggest that advection is not an identifiable driver in the data analyzed here, the methodology is flexible and would be able to identify such contributions should they arise in future experiments where directed motility effects are more prominent.

Other modeling approaches have used neural networks to perform data-driven inference on reaction and diffusion equations governing cell migration. In Variational System Identification, the equations learned are confined to the candidate library terms used, while neural networks can learn arbitrary relationships for equation parameters to best fit the data. Lagergren et al. demonstrated the power of this approach on the Jin et al. dataset [11] (also analyzed by Jin et al.), when they inferred density-dependent reaction-diffusion equations for cell migration [13]. They identified non-linear functions of density for diffusivity and reaction terms, and found that these functions vary with initial seeding density, consistent with our findings. There is one noteworthy difference between neural network-based approaches and Variational System Identification. In the case of cell migration, while the neural networks can learn arbitrary relationships between cell density and diffusivity or reaction (proliferation), there is no guarantee that physically realizable relationships are learnt, unless a number of physics-based constraints are built in. Variational System Identification, on the other hand, enables the modeler to restrict the candidate mechanisms and their mathematical forms to those that rigorously encode physical mechanisms. PINNs can infer diffusivity, advection, and reaction terms by treating them as unknown functions within the governing equations. However, unlike Variational System Identification, PINNs do not inherently promote parsimony, potentially leading to the identification of overly complex relationships rather than the most concise and mechanistically interpretable terms. A possible criticism of the Variational System Identification approach is that inference proceeds by model selection from a library of candidates, and not by a method of *de novo* discovery.

The Advection-Diffusion-Reaction (ADR) model provides a simple yet mechanistically meaningful continuum-scale representation of cell migration and proliferation. The advective and diffusive terms in the PDE naturally link to single-cell dynamics, where individual cell migration can be described as a stochastic process governed by an underlying stochastic differential equation. Meanwhile, the reaction term encapsulates cell division and death rates, making ADR models a versatile tool for inferring both collective- and single-cell-level behaviors from experimental data. Recently, delayed diffusion-reaction PDE models have been introduced to study scratch assay dynamics. [13,15,58,59] Delay terms in PDEs generally arise as phenomenological approximations, intended to capture latent biological processes that are difficult to model explicitly at the continuum scale. While such models provide descriptive frameworks for reconciling differences between low-density and high-density regimes, the precise relationship between delayed PDE formulations and stochastic single-cell dynamics is yet to be fully understood.

Delay differential equations also present difficulties in both inference and refinement. While progress has been made in the inference of delayed ordinary differential equations (ODEs) using techniques such as Delayed SINDy [60], and in delayed PDEs through approaches like Physics-Informed Neural Networks (PINNs) [13] and Maximum Likelihood Estimation (MLE) [59], computational methods for high-fidelity inference of these models remain limited. Our approach to PDE-constrained optimization leverages adjoint-based techniques to enable accurate parameter estimation in models. However, a major challenge in extending our framework to delayed PDEs is the scarcity of robust solvers that support automatic differentiation, which is essential for efficient gradient-based PDE-constrained optimization. Additionally, an extra layer of complexity is introduced by identifying and incorporating parameterized nonlinear delay kernels that can accurately capture history-dependent effects while remaining computationally tractable. Developing or integrating such solvers, alongside strategies for efficiently parameterizing and inferring nonlinear delay kernels, is a promising future direction in PDE-constrained optimization, and for our work.

The above difficulties around complete treatments of inference with delay-differential equations can be circumvented by including a restricted delay model within the VSI framework. In S2 Appendix we have explored a change-point treatment of delay differential equations, where we use ramp functions in time on the diffusive and reactive mechanisms. These approaches have been studied previously by Lagergren et al. [13] In our studies, these models show similar performance to our baseline VSI approach. However, this approach presents a simple extension to delay-differential equations pending the more extensive algorithmic developments that we have outlined above.

## 6. Conclusion

In summary, our study presents a computational pipeline that integrates Variational System Identification (VSI) for fast model discovery with PDE-constrained parameter optimization for refinement. This two-step approach enables the efficient inference of models describing collective cell migration and proliferation/death in wound healing assays. We benchmark this method by comparing inferred models with previous reaction-diffusion models applied to 1-D wound healing data, finding that our models are comparable in accuracy to the previously published, traditionally derived models. We next capture cell migration in 2-D wound healing assays using video microscopy, measuring the effect of trametinib on cell migration as a test case. We further find that the approach advanced here can be applied to 2-D wound healing to quantify effects of a targeted inhibitor on cell migration and density-dependent effects on proliferation. Our work demonstrates that this pipeline can be used to rapidly identify parsimonious models for cell migration and proliferation from scratch assays. In the future, we aim to address limitations in experiments that may not resolve length scales with sufficiently high cell densities for continuum-level predictions. In such cases, we plan to leverage the relationship between transport PDEs and Brownian motion-based stochastic differential equations to further inform the inference procedure when working with sparse data.

## Supporting information

S1 Appendix Confidence interval estimation. Derivation of local-quadratic uncertainty bounds and parameter estimates for the inferred PDE models.
(PDF)

S2 Appendix Delay mechanisms in migration. Detailed description of time-dependent migration responses modeled using delay functions *G*_1_-*G*_6_.
(PDF)
