# Supplementary material for: Inference of weak-form partial differential equations describing migration and proliferation mechanisms in wound healing experiments on cancer cells
Source: PLoS Comput Biol. 2025 Oct 28;21(10):e1013607. doi: 10.1371/journal.pcbi.1013607 (PMC12578354; doi:10.1371/journal.pcbi.1013607)
Supplement: S1 Appendix — Derivation of local-quadratic uncertainty bounds and parameter estimates for the inferred PDE models. (PDF) [file pcbi.1013607.s001.pdf]

S1 Appendix: Confidence interval estimation

We interpret the loss in Eq. (16a) in the main text as arising from an additive Gaussian error model

$$C^h(\boldsymbol{x},t) \;=\; {C'}^h(\boldsymbol{x},t;\,\boldsymbol{\theta}) \;+\; \varepsilon(\boldsymbol{x},t), \qquad \varepsilon(\boldsymbol{x},t) \overset{\text{i.i.d.}}{\sim} \mathcal{N}(0,\sigma'^2), \tag{S1}$$

this inspires the log-likelihood of the data given some parameters $\overline{\boldsymbol{\theta}}$ to be prescribed as

$$\log L(\boldsymbol{\theta}; C^h) \;=\; -\frac{1}{2\sigma^2}\left\|{C'}^h(\boldsymbol{x},t;\boldsymbol{\theta}) - C^h(\boldsymbol{x},t)\right\|^2_{L_2(\Omega\times[0,T])} \;+\; \text{const.}$$

Thus minimizer or $\ell(\overline{\boldsymbol{\theta}})$ in Eq. (16a) in the main text is same as the maximizer $\log L$. Moreover, we prescribe the σ as the minimized value of ℓ.

Let $H = \nabla^2_{\boldsymbol{\theta}}\ell(\overline{\boldsymbol{\theta}})\big|_{\boldsymbol{\theta}^{**}}$ be the Hessian of the reduced objective (assembled via second-order adjoint actions). The Taylor expansion of the log-likelihood near the Maximum Likelihood Estimate (MLE), $\boldsymbol{\theta}^{**}$, is given as:

$$\log L(\boldsymbol{\theta}; C^h) \approx \ell(\boldsymbol{\theta}^{**}) - \frac{1}{2\sigma^2}(\boldsymbol{\theta}-\boldsymbol{\theta}^{**})^\top H(\boldsymbol{\theta}^{**})\,(\boldsymbol{\theta}-\boldsymbol{\theta}^{**})$$

Applying Bayes' theorem with a uniform prior $\pi(\boldsymbol{\theta}) \propto 1$ and the approximated likelihood yields

$$p(\boldsymbol{\theta}\mid\text{data}) \;\propto\; L(\boldsymbol{\theta}) \;\propto\; \exp\left[-\frac{1}{2}(\boldsymbol{\theta}-\boldsymbol{\theta}^{**})^\top \mathbf{I}\,(\boldsymbol{\theta}-\boldsymbol{\theta}^{**})\right], \quad \mathbf{I} = H/\sigma^2 \tag{S2}$$

so the posterior has a Gaussian distribution with the mean described by the maximum a posteriori (MAP) estimate that coincides with the MLE. And, the covariance is given by the $\mathbf{I}^{-1}$. Marginal standard deviation of the i-th parameter is $\text{std}(\theta_i) \approx (I^{-1})_{ii} = \sigma\sqrt{H^{-1}_{ii}}$. Here the uncertainty in the inferred parameters can be written using the 95% confidence interval bound, estimated as $[\theta_i^{**} - 1.96\times\text{std}(\theta_i), \theta_i^{**} + 1.96\times\text{std}(\theta_i)]$. The results of this analysis for Jin's data are presented in Table A in S1 Appendix, and those for the Trametinib data are presented in Table B in S1 Appendix.

It is worth noting that the estimated variability in the constant diffusivity parameter is high. In many cases, the standard deviation of the diffusivity estimate exceeds its mean value, leading to negative lower bounds of the confidence interval, that fall outside the physically admissible range. This limitation arises because the method estimates uncertainty based on the curvature of the loss function near the optimal parameters, which may not accurately reflect the true posterior distribution when the likelihood is relatively flat along certain parameter directions, as highlighted in our sensitivity analysis.

Another important limitation of this approach is the assumption of homoscedastic Gaussian noise. This assumption may not hold in practice, particularly when the candidate PDE library is incomplete, thereby introducing aleatoric uncertainty and correlated noise. As seen in the Trametinib experiments, the higher optimal loss values are indicative of larger data variance relative to the model solution. This increased variance may result from the restricted set of candidate terms available for the PDE advection–reaction–diffusion model. Addressing these aleatoric uncertainties will likely require expanding the model space to better capture the underlying system dynamics, while still leveraging the methodology developed in this work.

Table A: Inferred parameter estimates θ_i^{**}, parameter uncertainties from the local quadratic assumption of the log-likelihood for each initial density conditions in the Jin's data. The index column refers to specific bases. Index 0: constant diffusivity; Index 1: first order concentration dependent diffusivity; Index 2: second order concentration dependent diffusivity; Index 6: first order cell growth; Index 7: quadratic cell growth.

**Initial density**	**Index**	θ_i^{**}	$\sqrt{H_{ii}^{-1}}$	σ	std(θ_i)
10000	0	7.93×10^0	5.00×10^5	7.88×10^{-5}	3.94×10^1
	1	8.97×10^3	1.62×10^2		1.28×10^{-2}
	6	2.79×10^{-2}	6.96×10^1		5.49×10^{-3}
12000	0	1.46×10^1	3.81×10^5	7.81×10^{-5}	2.98×10^1
	1	2.85×10^3	1.73×10^2		1.35×10^{-2}
	6	2.33×10^{-2}	5.44×10^1		4.25×10^{-3}
14000	0	4.69×10^2	4.92×10^6	6.44×10^{-5}	3.17×10^2
	1	2.49×10^{-1}	3.83×10^3		2.47×10^{-1}
	6	2.34×10^{-2}	7.38×10^1		4.75×10^{-3}
16000	0	3.58×10^2	3.28×10^6	6.54×10^{-5}	2.14×10^2
	2	1.26×10^2	2.40×10^0		1.57×10^{-4}
	6	2.34×10^{-2}	6.20×10^1		4.05×10^{-3}
18000 (3-term)	0	1.72×10^1	1.88×10^5	4.94×10^{-4}	9.27×10^1
	1	1.32×10^{-3}	9.35×10^1		4.62×10^{-2}
	6	1.55×10^{-2}	1.63×10^1		8.05×10^{-3}
18000 (4-term)	0	3.64×10^1	2.71×10^6	4.53×10^{-5}	1.23×10^2
	1	1.20×10^{-2}	3.89×10^3		1.77×10^{-1}
	6	9.53×10^{-2}	1.51×10^3		6.83×10^{-2}
	7	-6.97×10^1	1.34×10^6		6.10×10^1
20000	0	8.09×10^2	7.04×10^6	6.11×10^{-5}	4.30×10^2
	2	3.18×10^{-2}	7.02×10^0		4.29×10^{-4}
	6	1.75×10^{-2}	7.49×10^1		4.58×10^{-3}

Table B: Inferred parameter estimates θ_i^{**}, parameter uncertainties from the local quadratic assumption of the log-likelihood for each experimental condition of Trametinib in the wound healing experiment. The index column refers to specific bases. Index 0: constant diffusivity; Index 6: first order cell growth; Index 7: quadratic cell growth.

Conditions	Index	θ_i^{**}	$\sqrt{H_{ii}^{-1}}$	σ	std(θ_i)
	0	2.66×10^1	5.95×10^0		1.74×10^1
$10\mu m$	6	8.17×10^{-2}	1.44×10^{-2}	1.03×10^2	2.50×10^{-1}
	7	-5.27×10^1	9.64×10^0		1.68×10^2
	0	2.76×10^1	6.01×10^0		1.64×10^1
$5\mu m$	6	7.04×10^{-2}	1.38×10^{-2}	9.86×10^1	2.27×10^{-1}
	7	-4.41×10^1	9.41×10^0		1.54×10^2
	0	2.35×10^1	5.51×10^0		1.55×10^1
$1\mu m$	6	7.14×10^{-2}	1.39×10^{-2}	8.54×10^1	2.15×10^{-1}
	7	-4.56×10^1	9.66×10^0		1.50×10^2
	0	2.98×10^1	6.86×10^0		1.41×10^1
$500nm$	6	6.25×10^{-2}	1.41×10^{-2}	9.68×10^1	2.00×10^{-1}
	7	-4.02×10^1	1.03×10^1		1.45×10^2
	0	2.79×10^1	6.32×10^0		1.48×10^1
$100nm$	6	6.62×10^{-2}	1.40×10^{-2}	9.39×10^1	2.08×10^{-1}
	7	-4.12×10^1	9.73×10^0		1.45×10^2
	0	5.00×10^1	1.02×10^1		2.13×10^1
NT	6	1.31×10^{-1}	1.76×10^{-2}	2.18×10^2	3.75×10^{-1}
	7	-9.16×10^1	1.26×10^1		2.67×10^2
