# Supplementary material for: Inference of weak-form partial differential equations describing migration and proliferation mechanisms in wound healing experiments on cancer cells
Source: PLoS Comput Biol. 2025 Oct 28;21(10):e1013607. doi: 10.1371/journal.pcbi.1013607 (PMC12578354; doi:10.1371/journal.pcbi.1013607)
Supplement: S2 Appendix — Detailed description of time-dependent migration responses modeled using delay functions G1-G6. (PDF) [file pcbi.1013607.s002.pdf]

S2 Appendix: Delay mechanisms in migration

We introduced 6 delay forms, denoted by $G_1 - G_6$ (described as piecewise-linear forms at timepoints, $t/T = \{0, 0.25, 0.5, 0.75, 1\}$ and illustrated in S1 Fig). For each G_i, we optimize the diffusive, advective, and reactive parameters in a manner that preserves the linearity of the PDE model with respect to the parameters to be inferred. This allows the VSI procedure to retain its least-squares formulation, along with the associated computational efficiency.

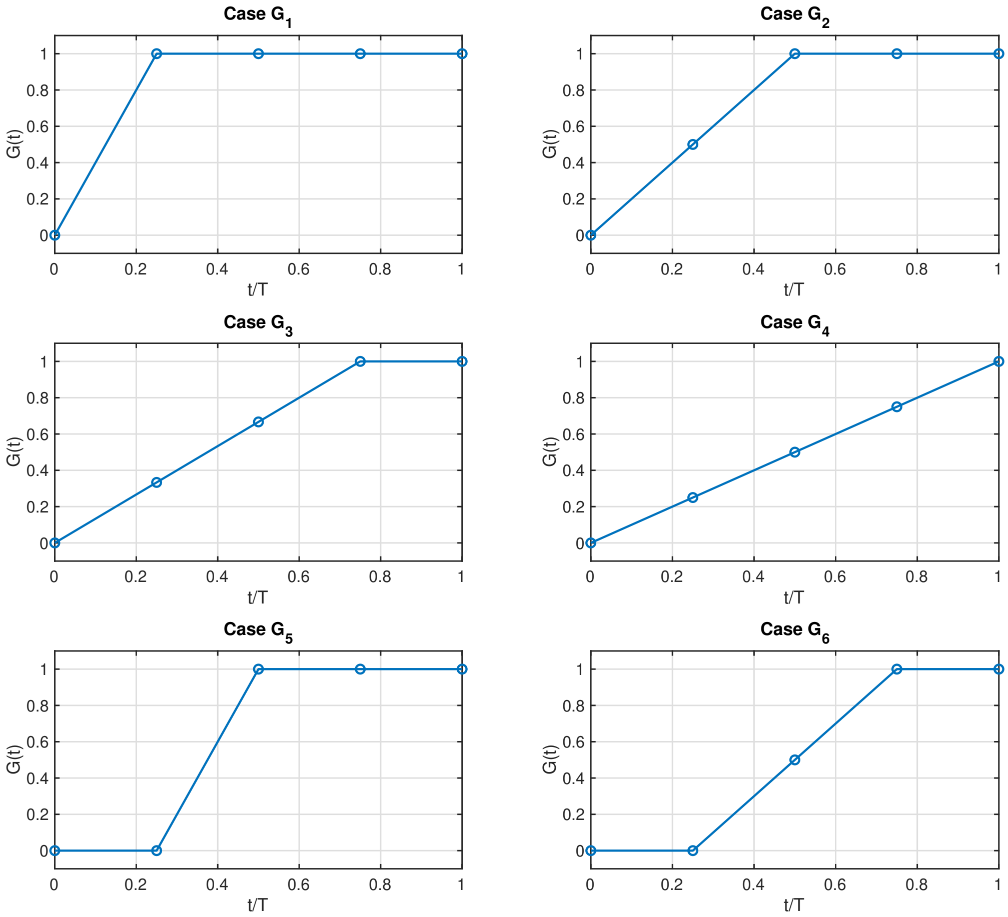

S1 Fig: Delay functions $G_{1-6}(t)$ described as piecewise linear functions

For each initial cell density, we performed VSI and PDE-constrained optimization using these $G(t)$ profiles. The resulting loss ℓ between the simulated model and the experimental data are reported in Table A in S2 Appendix.

Table A: L_2 losses between the simulated model and experimental data for six different $G(t)$ profiles and various initial densities.

Init. dens.	No delay	G_1	G_2	G_3	G_4	G_5	G_6
20000	6.11×10^{-5}	1.03×10^{-3}	1.06×10^{-3}	1.10×10^{-3}	9.02×10^{-5}	1.13×10^{-3}	8.94×10^{-5}
18000	4.94×10^{-4}	4.94×10^{-4}	5.15×10^{-4}	5.42×10^{-4}	5.50×10^{-4}	5.59×10^{-4}	6.02×10^{-4}
16000	6.54×10^{-5}	2.95×10^{-4}	3.14×10^{-4}	3.36×10^{-4}	3.46×10^{-4}	3.47×10^{-4}	3.85×10^{-4}
14000	6.44×10^{-5}	2.93×10^{-4}	3.10×10^{-4}	8.74×10^{-5}	3.43×10^{-4}	3.46×10^{-4}	1.16×10^{-4}
12000	7.81×10^{-5}	8.27×10^{-5}	5.42×10^{-5}	6.51×10^{-5}	1.15×10^{-4}	1.16×10^{-4}	1.42×10^{-4}
10000	7.88×10^{-5}	8.26×10^{-5}	9.02×10^{-5}	1.00×10^{-4}	1.06×10^{-4}	1.06×10^{-4}	1.24×10^{-4}

We first observe from Table A in S2 Appendix that the loss with no delay remains the lowest across most cases, with G_4 and G_6 showing comparably low losses for the 20000 initial density; all profiles $G_1 - G_6$ showing low losses comparable to the no-delay model for the 18000 initial density case; G_3 for the 14000 case; G_2 and G_3 for the 12000 case; and G_1 and G_2 for the 10000 case. This variation is possibly due to a suboptimal choice of the delay form, and to the simplification of applying the same delay profile to both the diffusive and reactive terms. Moreover, the dependence on initial density may be related to how quickly cells in different seeding conditions can sense the formation of the wound and respond to it. S2 Fig shows our inferred forward model with these delay functions compared to the ground truth data in each case.

This exercise nevertheless demonstrates that, when such a model with a prescribed family of $G(t)$ form is available, the present optimization framework can be directly applied to infer the corresponding parameters.

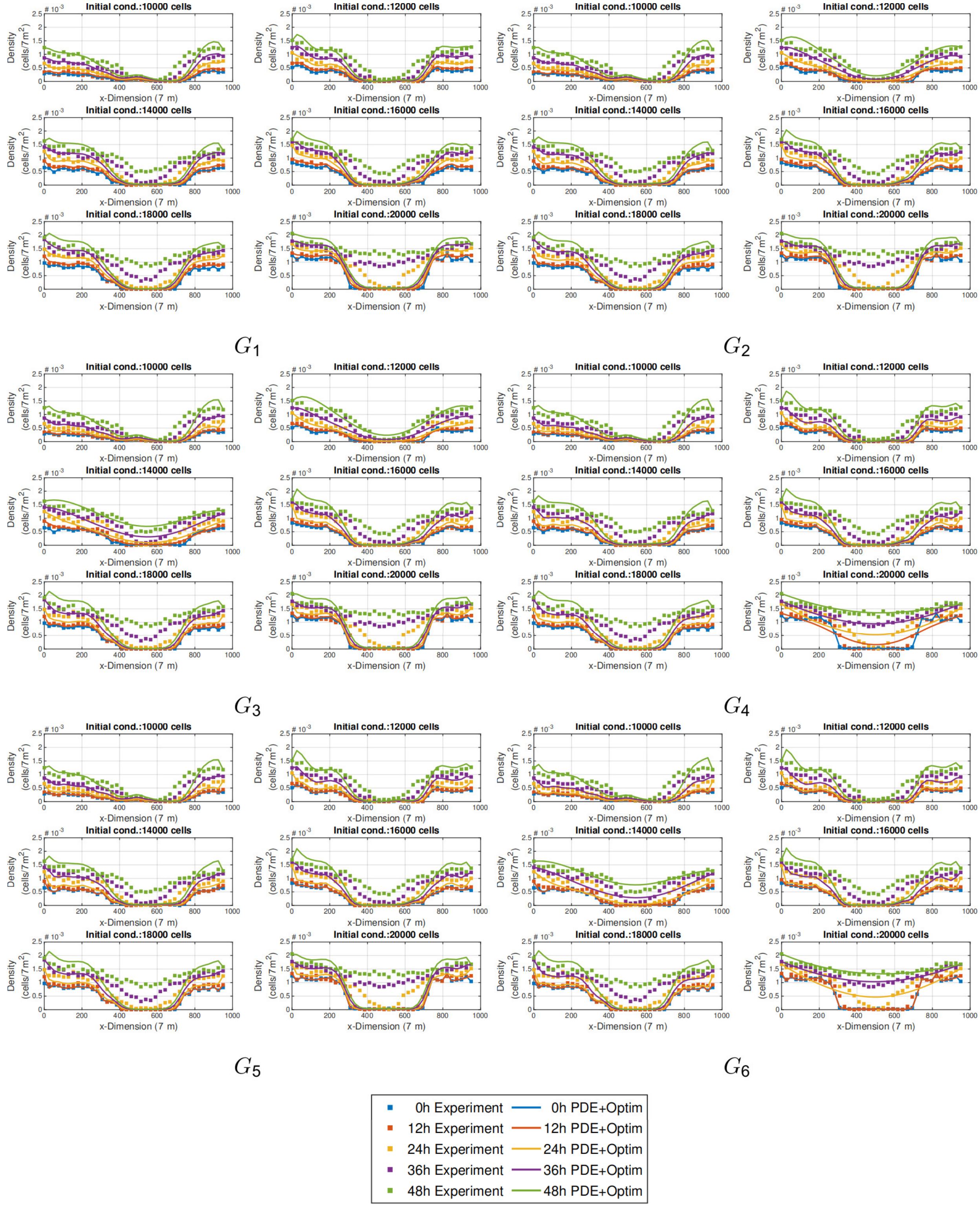

S2 Fig: Comparison of results for different delay settings.
